# The safety of a low-protein diet in older adults with advanced chronic kidney disease

**DOI:** 10.1093/ndt/gfae077

**Published:** 2024-03-27

**Authors:** Karin Windahl, Nicholas C Chesnaye, Gerd Faxén Irving, Peter Stenvinkel, Tora Almquist, Maarit Korkeila Lidén, Christiane Drechsler, Maciej Szymczak, Magdalena Krajewska, Esther de Rooij, Claudia Torino, Gaetana Porto, Fergus J Caskey, Christoph Wanner, Kitty J Jager, Friedo W Dekker, Marie Evans, the EQUAL study investigators, Karin Windahl, Nicholas C Chesnaye, Gerd Faxén Irving, Peter Stenvinkel, Tora Almquist, Maarit Korkeila Lidén, Christiane Drechsler, Maciej Szymczak, Magdalena Krajewska, Esther de Rooij, Claudia Torino, Gaetana Porto, Fergus J Caskey, Christoph Wanner, Kitty J Jager, Friedo W Dekker, Marie Evans

**Affiliations:** Division of Renal Medicine, Department of Clinical Intervention and Technology, Karolinska Institutet, Stockholm, Sweden; Division of Clinical Nutrition and Dietetics, Department of Orthopedics, Danderyds Hospital, Stockholm, Sweden; ERA Registry, Amsterdam UMC location University of Amsterdam, Medical Informatics, Amsterdam, The Netherlands; Amsterdam Public Health Research Institute, Quality of Care, Amsterdam, The Netherlands; Division of Clinical Geriatrics, Department of Neurobiology, Care Science and Society, Karolinska Institutet, Stockholm, Sweden; Division of Renal Medicine, Department of Clinical Intervention and Technology, Karolinska Institutet, Stockholm, Sweden; Division of Nephrology, Department of Clinical Sciences, Danderyds Hospital, Stockholm, Sweden; Division of Renal Medicine, Department of Clinical Intervention and Technology, Karolinska Institutet, Stockholm, Sweden; Department of Medicine, Division of Nephrology, University Hospital of Würzburg, Würzburg, Germany; Department of Nephrology and Transplantation Medicine, Wroclaw Medical University, Wroclaw, Poland; Department of Nephrology and Transplantation Medicine, Wroclaw Medical University, Wroclaw, Poland; Department of Clinical Epidemiology, Leiden University Medical Centre, Leiden, The Netherlands; 4CNR-IFC, Clinical Epidemiology and Physiopathology of Renal Diseases and Hypertension, Reggio Calabria, Italy; G.O.M., Bianchi Melacrino Morelli, Reggio Calabria, Italy; Department of Renal Medicine, North Bristol NHS Trust, Bristol, UK; Population Health Sciences, University of Bristol, Bristol, UK; Department of Clinical Research and Epidemiology, Comprehensive Heart Failure Center, University Hospital of Würzburg, Würzburg, Germany; ERA Registry, Amsterdam UMC location University of Amsterdam, Medical Informatics, Amsterdam, The Netherlands; Amsterdam Public Health Research Institute, Quality of Care, Amsterdam, The Netherlands; Department of Clinical Epidemiology, Leiden University Medical Centre, Leiden, The Netherlands; Division of Renal Medicine, Department of Clinical Intervention and Technology, Karolinska Institutet, Stockholm, Sweden

**Keywords:** age, CKD, malnutrition, nutrition, survival analysis

## Abstract

**Background:**

A low-protein diet (LPD) is recommended to patients with advanced chronic kidney disease (CKD), whereas geriatric guidelines recommend a higher amount of protein. The aim of this study was to evaluate the safety of LPD treatment in older adults with advanced CKD.

**Methods:**

The EQUAL study is a prospective, observational study including patients ≥65 years of age with an incident estimated glomerular filtration rate <20 ml/min/1.73 m^2^ in six European countries with follow-up through 6 years. Nutritional status was assessed by a 7-point subjective global assessment (SGA) every 3–6 months. Prescribed diet (g protein/kg of bodyweight) was recorded on every study visit; measured protein intake was available in three countries. Time to death and decline in nutritional status (SGA decrease of ≥2 points) were analysed using marginal structural models with dynamic inverse probability of treatment and censoring weights.

**Results:**

Of 1738 adults (631 prescribed LPD at any point during follow-up), there were 1319 with repeated SGA measurements, of which 267 (20%) decreased in SGA ≥2 points and 565 (32.5%) who died. There was no difference in survival or decrease in nutritional status for patients prescribed a LPD ≤0.8 g/kg ideal bodyweight {odds ratio [OR] for mortality 1.15 [95% confidence interval (CI) 0.86–1.55)] and OR for decrease in SGA 1.11 [95% CI 0.74–1.66]} in the adjusted models. In patients prescribed a LPD <0.6 g/kg ideal bodyweight, the results were similar. There was a significant interaction with LPD and older age >75 years, lower SGA and higher comorbidity burden for both mortality and nutritional status decline.

**Conclusions:**

In older adults with CKD approaching end-stage kidney disease, a traditional LPD prescribed and monitored according to routine clinical practice in Europe appears to be safe.

## Graphical Abstract

**Graphical Abstract:**
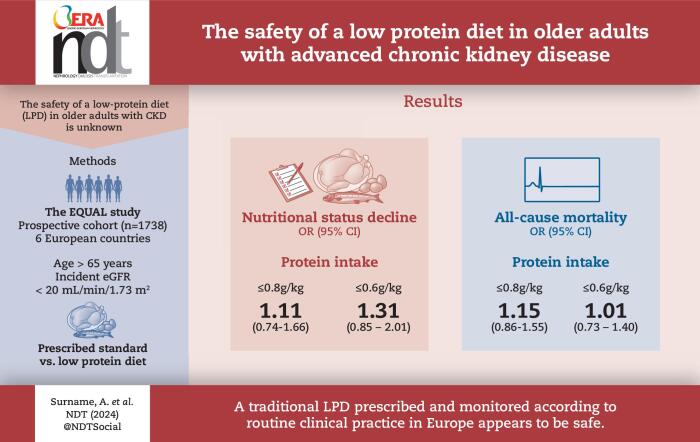

KEY LEARNING POINTS **What was known:**

- The challenges of nutritional care in older adults with advanced chronic kidney disease (CKD) are many and current nutritional guidelines are contradictory.
- CKD guidelines suggest the use of a low-protein diet (LPD), whereas geriatric guidelines recommend a higher amount of protein to reduce the risk of sarcopenia and malnutrition.

**This study adds:**

- This study suggests that LPD, prescribed and monitored according to routine clinical practice in Europe, is a safe treatment for older adults with CKD.
- In patients with a high-risk profile, such as the very old and those with a large comorbidity burden, the benefits of LPD should be weighed against the risks of accelerated nutritional status decline.

**Potential impact:**

- The results from this large, European study may contribute to improved nutritional management in older adults with CKD and underscore the importance of nutritional monitoring regardless of dietary regimen.

## INTRODUCTION

A low-protein diet (LPD) is recommended to patients with advanced chronic kidney disease (CKD) to delay kidney decline progression and improve quality of life [1]. A LPD needs to be prescribed under careful considerations to avoid protein energy wasting (PEW), since a decrease in appetite, development of a poor nutritional status and weight loss begin relatively early in the course of CKD [2–5].

Many of CKD patients are elderly; >50% of all European patients on maintenance dialysis treatment are >65 years of age [6, 7]. Older adults have several comorbid conditions and a high prevalence of poor nutritional status [3, 8]. Nutrition guidelines for older adults are contradictory. While CKD guidelines in general suggest the use of LPD in CKD stages 3–5, geriatric guidelines recommend a daily amount of at least 1.0 g protein/kg [9] to reduce the risk of sarcopenia and malnutrition. If dialysis treatment is initiated, the protein requirement increases. The recommended intake is 1.0–1.2 g/kg/day to maintain a stable nutritional status and to compensate for losses during the dialysis procedure [1]. The use of a LPD differs between countries and in regions in the same country [10, 11]. A LPD can be tailored and provided in several ways. The traditional LPD is based on mixed protein sources (0.6–0.8 g/kg/day) and often consists of regional cuisines. Very-low-protein diets (0.3–0.4 g/kg/day) should be supplemented with amino acid or ketoacid supplements to reach the requirements for essential amino acids [1]. Recently published studies showed potential benefits of plant-dominant LPDs in CKD [12, 13]. An adequate energy intake is essential regardless of the protein level or type of protein source. Usually patients who are prescribed a LPD are carefully monitored by nephrologists and dietitians, who also consider the total energy intake, assess signs of PEW and evaluate dietary adherence [10, 11, 14–16]. The challenges of nutritional care in older adults with CKD are many [17] and the Kidney Disease Outcomes Quality Initiative (KDOQI) nutrition guideline have raised concern about the safety of a LPD in older adults.

Very few studies have investigated the association between LPDs and changes in nutritional status and mortality in older adults with CKD at risk of malnutrition. The aim of this study was to evaluate the safety of LPD treatment in older adults with advanced CKD, approaching end-stage kidney disease. For this purpose, we used a large European inception cohort of carefully phenotyped patients with stages 4–5 CKD and >65 years of age with repeated follow-up visits up to 6 years in routine nephrology care.

## MATERIALS AND METHODS

### Study design and study population

The EQUAL study is a multicentre, prospective observational cohort study involving six European countries (Germany, Italy, The Netherlands, Poland, Sweden and the UK). Inclusion criteria are people >65 years of age with an incident estimated glomerular filtration rate (eGFR) <20 ml/min/1.73 m^2^ under nephrology care. The patients received routine medical care as provided by the nephrology clinics in each country; the study visits took place every 6 months until the eGFR decreased to <10 ml/min/1.73 m^2^, after which the interval was every 3 months. At each study visit, the nephrologist completed a questionnaire with extensive clinical data and information on the prescribed diet. All standardized data were collected repeatedly, including comorbidities, nutritional status assessed by a 7-point subjective global assessment (SGA), medication and routine blood and urine biochemistry. Patients were followed up to 6 years. A full description of the study protocol has been published elsewhere [18]. For this study, we included participants who had entered the study before 26 August 2020 and for whom we had information regarding prescribed diet (Supplementary Fig. S1). All the study participants signed a written informed consent and the EQUAL study was approved by the ethical review board in all participating countries.

### LPD

On every follow-up visit, the nephrologist indicated whether the patient was prescribed a protein restriction and the specified amount in g/kg bodyweight. In the main analysis, we defined any prescribed protein intake ≤0.8 g/kg bodyweight as a LPD. To evaluate adherence to the diet regime, we used measured urea appearance from 24-hour urinary collections (patients in Sweden, Italy and The Netherlands). The dietary protein intake was then calculated according to the Maroni formula [19] and was normalized to an adjusted body mass index (BMI) of 23 kg/m^2^ [1] for each period when the patient was prescribed a standard diet or LPD until they started kidney replacement therapy. To test the robustness of the results, we additionally used a lower cut-off level of ≤0.6 g protein/kg bodyweight as our definition of a LPD. To evaluate the outcomes stratified by dietary adherence we divided a period when a patient was prescribed a standard diet into either ‘standard diet—adherent’ if the measured protein intake was >0.8 g/kg or ‘spontaneously low protein intake’ if the measured protein intake was ≤0.8 g/kg. The period when the patient was prescribed a LPD was categorized similarly into ‘LPD ≤0.8 g/kg adherent’, ‘LPD ≤0.8 g/kg non-adherent’ and ‘LPD ≤0.6 g/kg adherent’.

### Outcomes

Nutritional status was assessed during every follow-up visit with the 7-point SGA [20], where a score of 6–7 corresponds to good nutritional status, 3–5 is moderate malnutrition and <3 is severe malnutrition. To minimize the risk of misclassification, we regarded a decrease in SGA of at least 2 points as a decline in nutritional status. Vital status and cause of death were collected as part of the study protocol.

### Covariates

Information regarding demographics (age, sex, country), clinical information (primary renal disease, comorbidity), socio-economic status (level of education, marital status) and lifestyle (alcohol intake, smoking habits) were collected at baseline, whereas laboratory values [haemoglobin, plasma albumin, sodium, potassium, phosphorous, calcium, parathyroid hormone, urea, standard bicarbonate, eGFR (using the Chronic Kidney Disease Epidemiology Collaboration creatinine equation) [21] and cholesterol] and clinical data [blood pressure (BP), BMI, kidney replacement therapy] were collected during the entire follow-up.

### Statistical analyses

The covariates were described as means, medians and proportions stratified by LPD according to their underlying distribution and compared by non-parametric statistics. Education was categorized into four classes (low, intermediate, high and other); smoking habits were categorized as current smoker, former smoker or never smoker; and alcohol consumption was categorized into four categories based on the average number of units of alcohol per week. Patients with no information were categorized into a separate category. Patients were followed from baseline until the end of the follow-up period or death. We performed two separate analyses for our main outcomes of a decrease in SGA and mortality. For the survival analysis we included all patients with information regarding the prescribed diet. Once a patient had started a LPD, the patient remained in that group until the end of follow-up (intention to treat). Since we hypothesized that the potential effects of diet not only could influence the risk of immediate outcomes, but also future risk, we followed up regardless of whether the patient started kidney replacement therapy or not. However, the probability of treatment was only computed for the patients as long as they were not on dialysis. For the SGA analysis we excluded patients with fewer than two SGA measurements and followed them until the date of a decrease in SGA of at least 2 units from baseline or death.

Marginal structural survival models (pooled logistic regression models) were used to investigate the relationship between the treatment and our two outcomes [22, 23]. The stabilized inverse probability weights for receiving or not receiving treatment with a LPD were computed over the follow-up period using logistic regression models including information on age, sex, country, all relevant comorbidities, Charlson comorbidity index score, level of education, marital status, alcohol, smoking, BP and laboratory measurements (haemoglobin, albumin, potassium, urea, phosphate, eGFR) at baseline and all time-updated laboratory measurements and BP measurements. For the mortality analyses we additionally included SGA at baseline and over time. To account for informative censoring, we then computed the stabilized censoring weights similarly. The stabilized weights were centred around 1.0 with a low standard deviation; there were no extreme weights, and no truncation was therefore applied. To account for non-linear effects, we modelled time as a natural cubic spline with three knots. The outcome model included the baseline covariates described above. The interactions between prescribed diet and several pre-defined subgroups [age >75 and <75 years, high (≥6) versus low comorbidity score, diabetes mellitus, sex and normal versus lower (<6) SGA] were investigated and if statistically significant (*P* < .05) we performed stratified analyses. Missing values are reported in Supplementary Table S1.

We evaluated risk associated with adherence to a LPD in a restricted subgroup analyses where information on measured protein intake was available. For these analyses we first compared patients with a ‘standard diet—adherent’ to ‘LPD ≤0.8 g/kg adherent’ and to ‘LPD ≤0.8 g/kg non-adherent’. We subsequently compared patients with a ‘spontaneously low protein intake’ to patients with ‘LPD ≤0.8 g/kg adherent’ and ‘LPD ≤0.8 g/kg non-adherent’. For descriptive purposes, we additionally compared ‘spontaneously low protein intake’ to ‘standard diet—adherent’ using time-dependent Cox proportional hazards models and cumulative incidence curves adjusting for age, sex, eGFR and country.

We also performed several sensitivity analyses. First, we repeated both main outcomes and subgroups for patients prescribed ≤0.6 g protein/kg bodyweight. Second, we repeated the analyses after imputing the missing laboratory values with everyone's mean, followed by the population mean. Third, we analysed the measured protein intake by actual weight instead of ideal bodyweight. All analyses were performed with Stata 15 (StataCorp, College Station, TX, USA).

## RESULTS

### Patient characteristics

In total, we included 1738 individuals, of which 1319 had at least two SGA measurements and were retained in the analysis of SGA decline (Supplementary Fig. S1). Patient characteristics, stratified on the prescribed diet regimen, are presented in Table 1, and baseline characteristics for patients by measured protein intake are presented in Supplementary Table S2. The median age was 76 years and 65% were male. At baseline, 737 patients (43%) had a good nutritional status, 953 (55%) were moderately malnourished and 34 (2%) were classified with severe malnutrition. Over a median follow-up time of 2.1 years [interquartile range (IQR) 0.9–6.5], 500 started dialysis and 75 individuals were kidney transplanted.

**Table 1:** Patient characteristics at baseline stratified by prescription of a LPD during any time over the follow-up period.

	Standard diet	Low protein diet	
Characteristics	(*n* = 1107)	(*n* = 631)	*P*-value
Female, *n* (%)	413 (37)	191 (30)	.01
Age (years), median (IQR)	76 (71–81)	76 (70–81)	.65
Country, *n* (%)			<.001
Germany	146 (13)	8 (1)	
Italy	124 (11)	289 (46)	
Netherlands	119 (11)	144 (23)	
Poland	93 (8)	9 (1)	
Sweden	124 (11)	181 (29)	
UK	501 (45)	0 (0)	
Primary renal disease, *n* (%)			.86
Glomerular disease	91 (9)	34 (8)	
Tubulointerstitial disease	80 (8)	40 (9)	
Systemic disease	21 (2)	5 (1)	
Diabetes	197 (20)	88 (20)	
Hypertension, renovascular diseases	304 (33)	175 (39)	
Hereditary disease	30 (3)	9 (2)	
Other specified disorders	46 (5)	14 (3)	
Unknown	186 (20)	80 (18)	
Clinical data			
eGFR (ml/min/1.73 m^2^), mean (SD)	17.6 (5.6)	16.7 (5.2)	<.001
Systolic BP (mmHg), median (IQR)	141 (130–158)	140 (126–154)	.01
Diastolic BP (mmHg), median (IQR)	73 (66–80)	74 (68–80)	.40
BMI (kg/m^2^), medina (IQR)	28.3 (25–32)	27.2 (24–31)	<.001
SGA overall score, mean (SD)	5.9 (1.0)	6.1 (1.0)	<.001
Supplement amino acids, *n* (%)	4 (0.4)	44 (7)	.02
Start kidney replacement therapy, *n* (%)	300 (27)	200 (32)	.11
Laboratory measurements, mean (SD)			
Haemoglobin (g/l)	129 (17)	130 (16)	.17
Sodium (mmol/l)	140 (3)	140 (3)	.13
Potassium (mmol/l)	4.7 (0.6)	4.6 (0.6)	.01
Phosphate (mmol/l)	1.3 (0.3)	1.3 (0.3)	.98
Urea (mmol/l)	20.1 (8.2)	22.6 (11.0)	<.001
Albumin (g/l)	38 (6.0)	37 (5.5)	.01
Cholesterol (mmol/l)	4.6 (1.4)	4.5 (1.2)	.87
Comorbidity, *n* (%)			
Charlson comorbidity index, mean (SD)	7.1 (1.8)	7.2 (2.0)	.14
Diabetes mellitus	443 (40)	266 (42)	.53
Cerebrovascular disease	159 (14)	99 (16)	.28
Coronary artery disease	351 (32)	202 (32)	.21
Malignancy	211 (19)	139 (22)	.53
Heart failure	180 (16)	119 (19)	.41
Education, *n* (%)			
Low	266 (32)	160 (29)	.31
Intermediate	407 (49)	267 (49)	
High	112 (13)	99 (18)	
Other	49 (6)	20 (4)	
Marital status, *n* (%)			.05
Married/partner	515 (62)	371 (68)	
Divorced/widowed/single	318 (38)	174 (32)	
Lifestyle			
Smoker			.87
Never	306 (37)	206 (38)	
Current	69 (8)	50 (9)	
Former	452 (55)	283 (53)	
Alcohol consumption			.81
None	475(57)	299 (56)	
1–4 standard units/week	182 (22)	129 (24)	
>4–7 standard units/week	69 (8)	49 (9)	
>7 standard units/week	101 (12)	58 (11)	

### Diet

**Figure 1:**
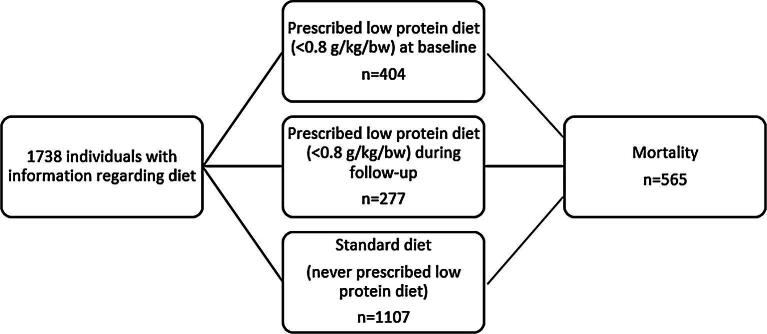
Description of the exposure, prescribed diet.

Among the 1738 patients, 631 patients (36%) were prescribed a LPD at some point during the follow-up (Fig. 1). Of these patients, 363 were prescribed a LPD with ≤0.6 g/kg bodyweight/day, of which 38 patients were prescribed a very-low-protein diet (0.3–0.4 g/kg). In total, 404 (23%) had a LPD at the first visit when included in the study.

### Decline in nutritional status

During follow-up, 268 (20%) patients declined in the 7-point SGA by ≥2 points and 342 (26%) died before they reached the SGA endpoint. The crude incidence rate over 4 years per 100 person-years was 7.7 (IQR 6.7–8.9) in those with a standard diet and 10.9 (IQR 9.2–13.2) in individuals prescribed a LPD (≤0.8 g/kg) (Table 2). In the group prescribed ≤0.6 g/kg, the incidence rate was 9.8 (IQR 7.6–12.8) (Table 2). The adjusted odds ratio (OR) for a decrease in the 7-pooint SGA was 1.11 (IQR 0.74–1.66) in those prescribed a LPD ≤0.8 g/kg compared with a standard diet. There was a statistically significant interaction (*P* < .05) between the risk of nutritional status decline and LPD treatment for age and comorbidity, while the interaction term was not consistent for LPD 0.8 g/kg or ≤0.6 g/kg for sex and diabetes (Supplementary Table S3). The results indicated a higher risk of a SGA decrease in those >75 years of age and a higher comorbidity score, but the individual subgroups did not reach statistical significance.

**Table 2:** Risk of decrease in SGA in older adults prescribed a LPD according to prescription and measured protein intake over the follow-up period.

Prescribed diet (*n* = 1319)	Number of events/person-years	Incidence rate over 4 years per 100 person-years (IQR)	Unadjusted OR (95% CI)	Adjusted OR^a^ (95% CI)
Standard diet (>0.8 g protein/kg) (*n* = 1004)	196/2529	7.7 (6.7–8.9)	Ref	Ref
LPD ≤0.8 g/kg (*n* = 483)	117/1065	10.9 (9.2–13.2)	1.43 (1.11–1.86)	1.11 (0.74–1.66)
LPD ≤0.6 g/kg (*n* = 288)	56/569	9.8 (7.6– 12.8)	1.65 (1.23–2.22)	1.31 (0.85–2.01)
Measured diet (*n* = 528)		Incidence rate per 100 person-years (IQR)	Unadjusted RR (95% CI)	Adjusted RR^b^ (95% CI)
Standard diet, adherent (*n* = 227)^b^	30/453	6.6 (4.6–9.5)	ref	ref
LPD ≤0.8 g/kg, adherent (*n* = 194)^b^	37/420	8.8 (6.4–12.1)	1.77 (0.85–3.72)	1.67 (0.75–3.77)
LPD ≤0.8 g/kg, non-adherent (*n* = 219)^b^	47/505	9.3 (7.0–12.4)	1.37 (0.70–2.66)	0.95 (0.50–1.78)
Standard diet, spontaneously low protein intake (*n* = 139)^b^	27/266	10.1 (7.0–14.8)	Ref	Ref
LPD ≤0.8 g/kg, adherent (*n* = 164)^b^	33/374	8.8 (6.3–12.4)	0.82 (0.40–1.67)	0.77 (0.30–2.01)^c^

Ref; reference; RR: relative risk.

a Dynamic inverse probability weighted analysis. Probability of treatment weights and stabilized censoring weights included baseline sex, country, Charlson comorbidity index, diabetes, ischaemic heart disease, peripheral arterial disease, heart failure, cancer, education, marital status, smoking, alcohol intake, time-updated age, eGFR, BMI, haemoglobin, albumin, urea, potassium, phosphate, systolic and diastolic BP and time (natural cubic spline with 3 knots). The outcome model further included age at inclusion, sex, country, comorbidity (diabetes, ischaemic heart disease, stroke, heart failure, peripheral arterial disease), eGFR at inclusion, SGA, smoking, BMI, haemoglobin and albumin at inclusion and time-varying kidney replacement therapy.

b Time-varying exposure resulting in a patient that may count in several categories.

c Country and level of education dropped due to failure of convergence.

**Figure 2:**
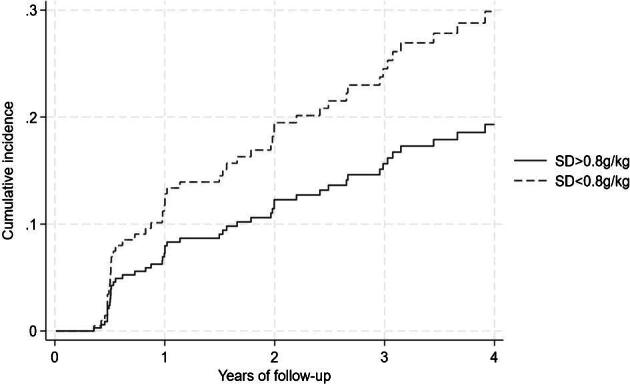
Cumulative incidence for decrease in the 7-point SGA in patients with spontaneously low protein intake (prescribed standard diet, but low protein intake <0.8 g/kg/day) and a standard diet with standard intake of protein (>0.8 g/kg/day).

Among the subgroup with measured protein intake there were 528 individuals from three countries. The SGA decreased by >2 points with an incidence of 6.6 per 100 person-years [95% confidence interval (CI) 4.6–9.5] in those with ‘standard diet—adherent’ as compared with patients with a spontaneously low protein intake [incidence 10.1 per 100 person-years (95% CI 7.0–14.8)]. As compared with patients with ‘standard diet—adherent’, the adjusted OR was 1.67 (95% CI 0.75–3.77) for a LPD ≤0.8 g/kg adherent and 0.95 (95% CI 0.50–1.78) for LPD ≤0.8 g/kg non-adherent. Comparison with patients with a spontaneously low protein intake, the OR was 0.77 (95% CI 0.30–2.01) for patients with LPD ≤0.8 g/kg adherent. The cumulative incidence of SGA decrease for patients with a spontaneously low protein intake versus standard diet adherent is illustrated in Fig. 2. As compared with a standard diet adherent, a spontaneously low protein intake was associated with a SGA decrease in the unadjusted analyses [hazard ratio (HR) 1.81 (95% CI 1.03–3.2)], but not in analyses adjusted for age, sex, eGFR and country [HR 1.54 (95% CI 0.82–2.87)] (data not shown).

### Mortality

During follow-up, 565 (32.5%) patients died. The mortality rate (crude incidence rate per 100 person-years) was 11.2 (95% CI 10.1–12.4) in those with a standard diet, 12.5 (95% CI 10.8–14.4) in those prescribed a LPD ≤0.8 g/kg and 12.3 (95% CI 10.1–14.9) in those prescribed a LPD ≤0.6 g/kg (Table 3). As compared with a standard diet, the adjusted OR for all-cause mortality was 1.15 (95% CI 0.86–1.55) for those prescribed a LPD ≤0.8 g/kg and 1.01 (95% CI 0.73–1.40) for a LPD ≤0.6 g/kg (Table 3). There was a statistically significant interaction (*P* < .05) between age, comorbidity and 7-pooint SGA, suggesting a higher risk for patients >75 years of age treated with LPD, a higher Charlson comorbidity score (>6 points) or lower nutritional status (7-point SGA <6). However, none of the risk estimates for the individual subgroups reached statistical significance (Supplementary Table S3).

**Table 3:** Mortality risk in older adults prescribed a LPD, according to prescription and measured protein intake over the follow-up period.

		All-cause mortality		
Prescribed diet (*n* = 1738)	Number of events/person-years	Crude incidence rate per 100 person-years (IQR)	Unadjusted OR	Adjusted OR^a^
Standard diet (*n* = 1329^b^)	368/3298	11.2 (10.1–12.4)	Ref	Ref
LPD ≤0.8 g/kg (*n* = 631^b^)	191/1529	12.5 (10.8–14.4)	1.10 (0.93–1.31)	1.15 (0.86–1.55)
LPD ≤0.6 g/kg (*n* = 363^b^)	99/808	12.3 (10.1–14.9)	1.06 (0.85–1.31)	1.01 (0.73–1.40)
Restricted analysis according to measured protein intake (*n* = 778)^b^		All-cause mortality		
Standard diet, adherent (*n* = 280)	47/593	7.9 (5.9–10.6)	Ref.	Ref.
LPD ≤0.8 g/kg, adherent (*n* = 229)	68/669	12.4 (9.8–15.7)	1.49 (1.03–2.16)	0.81 (0.46–1.43)
LPD ≤0.8 g/kg, non-adherent (*n* = 280)	77/697	11.0 (8.8–13.8)	1.33 (0.92–1.92)	0.97 (0.60–1.58)
Standard diet, spontaneously low protein intake (*n* = 169)	41/361	11.4 (8.4–15.4)	Ref.	Ref.
LPD ≤0.8 g/kg, adherent (*n* = 180)	56/464	12.1 (9.3–15.7)	1.04 (0.71–1.53)	1.23 (0.68–2.20)

Ref: reference.

a Dynamic inverse probability of treatment weights and stabilized censoring weights included baseline sex, country, Charlson comorbidity index, diabetes, ischaemic heart disease, peripheral arterial disease, heart failure, cancer, education, marital status, smoking, alcohol intake, time-updated age, eGFR, SGA, BMI, haemoglobin, albumin, urea, potassium, phosphate, systolic and diastolic BP and time (natural cubic spline with 3 knots). The outcome marginal structural model further included age at inclusion, sex, country, comorbidity (diabetes, ischaemic heart disease, stroke, heart failure, peripheral arterial disease), eGFR at inclusion, SGA, smoking, BMI, haemoglobin and albumin at inclusion and time-varying kidney replacement therapy.

b Time-varying exposure resulting in a patient that may count in several categories.

In the restricted analysis according to measured protein intake (*n* = 778), the adjusted OR was 0.81 (95% CI 0.46–1.43) in patients with a LPD ≤0.8 g/kg adherent and 0.97 (95% CI 0.60–1.58) for patients with a LPD ≤0.8 g/kg non-adherent. The adjusted mortality rates were similar in those with a spontaneously low protein intake and a LPD ≤0.8 g/kg adherent. Sensitivity analyses where missing data were imputed and according to measured protein intake per actual bodyweight demonstrated similar results as the main analyses (Supplementary Table S4).

## DISCUSSION

In this large European cohort with older CKD adults, we did not find an increased risk of nutritional status decline or mortality in patients prescribed a LPD as compared with a standard diet. However, the results suggested that there may be differences in safety depending on underlying patient characteristics. Although the individual subgroups did not reach statistical significance, there was an interaction between the risk for both mortality and nutritional status decline, indicating a higher risk with a LPD in patients >75 years of age and those with a higher comorbidity burden. Our results further suggested that patients with a spontaneously low protein intake had a higher risk of a decline in nutritional status compared with patients who adhered to a standard diet.

There are limited data on the long-term use of a LPD in elderly patients with CKD and its association with safety outcomes. Brunori *et al*. [24] studied mortality in a prospective, randomized controlled trial that included 112 patients >70 years of age. They compared individuals with a very-low-protein diet and patients on dialysis over a 48-month follow-up period. The study concluded that a very-low-protein diet was safe and postponed dialysis treatment by a median of 10.7 months. In a National Health and Nutrition Examination Survey study, a high protein intake was associated with mortality in people with an eGFR <60 ml/min/1.73 m^2^ and a mean age of 72 years, whereas lower levels of protein intake were not associated with death [25]. Hung *et al*. [26] performed a study in which 103 older adults with an eGFR <45 ml/min/1.73 m^2^ and a LPD or standard diet were followed up to 1 year, concluding that although BMI decreased progressively, muscle mass was preserved according to bioimpedance measurements. In our subgroup analyses we found that patients on a standard diet with a spontaneously low protein intake, which often is the result of a diminished appetite, had the highest rate of decline in nutritional status, although the analyses failed to reach statistical significance. These results align with other observations where a spontaneous reduction in protein intake without careful monitoring of energy intake has been reported as harmful [27].

Usually patients prescribed a LPD are carefully monitored by the nephrologist and dietitian, who also consider the total energy intake and assess clinical signs of PEW [10]. Even if the patients do not completely adhere to the prescribed diet, the nutritional counselling and monitoring itself might be beneficial. In a study by Perrez-Torres *et al*. [28], a nutritional education program during the pre-dialysis phase showed positive effects on nutritional status, decreased hospital admissions and increased survival in patients starting dialysis. According to KDOQI guidelines, it is important to consider in what context the LPD is initiated [1]. If the patients are metabolically instable or suffer from acute disease, a LPD should not be prescribed. In line with these recommendations, we observed important interactions for older versus younger age and higher versus lower comorbidity burden. For mortality, there was also an interaction for baseline SGA assessment. Although none of the point estimates of these subgroups reached the significance level, possibly due to the sample size, the results call for caution in treating patients >75 years of age, with a higher comorbidity burden and a lower SGA score with LPD.

The major strength of this study is the large population with incident advanced CKD from six countries with extensive, prospectively collected, repeated clinical data, making the results generalizable to the clinical practice of nephrology care in Europe. Furthermore, the patients in our study were included when their eGFR dropped below the pre-defined level of 20 ml/min/1.73 m^2^, thus minimizing the risk of survivor bias. The use of rich, prospectively collected information made it possible to analyse the data using marginal structural models with dynamic inverse probability weights. This type of analysis considers not only informative censoring, but also time-varying confounding. The decision to start a patient on a LPD is often influenced by current kidney function and interlinked uraemic symptoms, metabolic disturbances and nutritional status. Since these factors also influence future prognosis, time-varying confounding (or reverse causation) will be present unless it is considered in the analyses.

Another strength is our use of a repeated 7-point SGA to evaluate nutritional status. There are several methods to measure dietary adherence in CKD [1]. Subjective approaches such as food diaries and food frequency questionnaires are often used. In our study we used a combination of information of prescribed diet directly from the nephrologists’ questionnaire and calculations based on a repeated urea nitrogen levels from 24-hour urine collections, which are regarded as an objective, relatively unbiased method to assess protein intake. Since there is no international standard on which bodyweight should be used when prescribing protein level (real bodyweight regardless of BMI or adjusted body weight), we standardized our measured protein intake to a normal bodyweight with a BMI of 23 kg/m^2^. However, changing the estimations to real bodyweight did not meaningfully change the results.

Our study has also various limitations that should be addressed. As in all observational studies, we cannot confer causality, and although we have used all available data for adjustment in our models, there may still be residual confounding present. Another limitation is that the time points for collecting information on prescribed diet were fixed and the actual start of changes in a prescribed diet for the individual patient may have occurred between two study visits. However, in our subgroup analyses with measured protein intake, this should have been accounted for at least to some extent since measurements were repeated. Furthermore, we examined the protein intake and not the source of the proteins. Recent data suggest that protein from red meat and fish may have different effects on the progression of CKD [29]. Furthermore, renal care and the routine of nutritional management may differ between participating countries. By tradition, Italy, Sweden, and The Netherlands use a LPD to treat CKD patients, whereas Poland, Germany and the UK seldom do. When prescribing a LPD, the recommendation is to increase the intake of fat and carbohydrates to maintain the energy balance. However, the procedures for nutritional counselling and evaluating the energy and nutrient intake in the clinical setting may differ between the participating countries and are not standardized in the study protocol.

In conclusion, this study suggests that a traditional LPD prescribed and monitored according to routine clinical practice in Europe is a safe treatment for older adults with CKD. In people with a higher risk profile, such as the very old and those with a large comorbidity burden, the benefits of a protein-restricted diet should be carefully weighed against the potential risks of accelerated nutritional status decline. Since our study further suggests that a spontaneously low protein intake is associated with higher risk of nutritional status decline compared with adherence to a standard diet, our results further underscore the importance of monitoring the nutritional status and diet over time regardless of the dietary regimen.

## Supplementary Material

gfae077_Supplemental_File

## Data Availability

The data underlying this article are sensitive health data and cannot be shared publicly for privacy reasons. The data will be shared upon reasonable request to the corresponding author.
