# Supplementary material for: The safety of a low-protein diet in older adults with advanced chronic kidney disease
Source: Nephrol Dial Transplant. 2024 Mar 27;39(11):1867–75. doi: 10.1093/ndt/gfae077 (PMC11648958; doi:10.1093/ndt/gfae077)
Supplement: gfae077_Supplemental_File [file gfae077_Supplemental_File.pdf]

SUPPLEMENT ON-LINE

The safety of a low protein diet in older adults with advanced chronic kidney disease.

Authors: Karin Windahl, Nicholas C Chesnaye, Gerd Faxén Irving, Peter Stenvinkel, Tora Almquist, Maarit Korkeila Lidén, Christiane Drechsler, Maciej Szymczak, Magdalena Krajewska, Esther de Rooij, Claudia Torino, Gaetana Porto, Fergus J Caskey, Christoph Wanner, Kitty J Jager, Friedo W Dekker, Marie Evans, and the EQUAL study investigators

CONTENT	PAGE
**Figure S1**. Flow-chart inclusion	2
**Table S1**. Missing data at baseline	3
**Table S2.** Baseline characteristics of individuals with measured protein intake.	4
**Table S3.** Adjusted risk of mortality and decline in subjective global assessment associated with a prescribed low-protein diet, according to subgroups.	6
**Table S4.** Sensitivity analyses for the risk of mortality associated with a low protein diet	8

Figure S1. Flow-chart inclusion

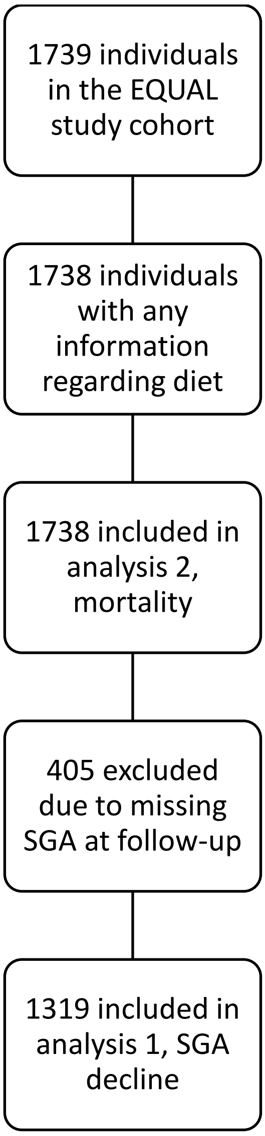

Table S1 Missing data at baseline

	**All** (n=1738)<br><br>Numbers (%)
Sex	0
Age	0
Country	0
eGFR mL/min/1.73²	63 (3.6)
Blood pressure mmHg	83 (4.8)
Body Mass Index kg/m²	150 (8.6)
Hb	79 (4.5
Potassium	42 (2.4)
Sodium	85 (5.0)
Phosphate	151 (8.7)
Urea	114 (6.6)
Albumin	215 (15.8)
SGA overall score	240 (13.8)
Charlson Comorbidity score	42 (2.4)
Diabetes	44 (2.5)
Cerebrovascular disease	53 (3.1)
Coronary artery disease	78 (4.5)
Malignancy	59 (3.4)
Heart failure	89 (5.2)
Education	345 (19.9)
Marital status	347 (20.0)
Smoking habits	402 (23.1)
Alcohol consumption	388 (22.3)

Table S2 Baseline characteristics* of individuals (n=778) with measured protein intake

	**Standard diet measured intake >0.8g/kg**	**Standard diet measured intake <0.8g/kg**	**Low protein diet measured intake >0.8g/kg***	**Low protein diet measured intake <0.8g/kg***
Female	49 (25)	44 (39)	79 (28)	72 (31)
Age years	73.8 (70-78)	76 (71-80)	76 (70-81)	75 (69-80)
Country n, %				
Germany	17 (1)	9 (8)	3 (1)	1 (1)
Italy	53 (17)	37 (32)	142 (51)	104 (45)
Netherlands	33 (27)	17 (15)	33 (12)	42 (18)
Poland	1 (1)	2 (2)	-	-
Sweden	88 (46)	48 (42)	102 (36)	82 (36)
United Kingdom	1 (9)	1 (1)	-	-
Primary Renal Disease n, %				
Glomerular disease	12 (11)	8 (12)	17 (10)	12 (8)
Tubulointerstitial disease	6 (6)	4 (6)	14 (8)	11 (8)
Systemic disease	4 (4)	1 (1)	2 (1)	3 (2)
Diabetes	24 (23)	13 (20)	38 (21)	33 (23)
Hypertension, renovascular diseases	32 (30)	28 (42)	58 (33)	57 (39)
Hereditary diseases	2 (2)	4 (6)	3 (2)	4 (3)
Other specified disorders	4 (4)	2 (3)	7 (4)	4 (3)
Unknown	21 (20)	6 (9)	38 (21)	21 (14)
Clinical data				
eGFR mL/min/1.73^2	18.6 (6.7)	16.0 (5.8)	17.0 (4.8)	16.3 (5.2)
Systolic blood pressure mmHg	140 (130-156)	141 (130-154)	140 (129-153)	142 (127-155)
Diastolic blood pressure mmHg	74 (67-83)	72 (66-80)	75 (70-81)	75 (11.8)
Body Mass Index kg/m^2	29 (25-32)	26 (23-29)	28 (25-31)	27 (24-31)
SGA overall score	6.2 (0.9)	5.6 (1.3)	6.2 (0.9)	6.0 (1.1)
Supplement amino acids	4 (0.4)	2 (2)	23 (8)	25 (11)
Laboratory measurements				
Hemoglobin mg/dL	133 (16)	129 (16)	131 (16)	131 (17)
Sodium mmol/L	141 (3)	140 (4)	140 (3)	140 (4)
Potassium mmol/L	4.5 (0.6)	4.5 (0.6)	4.6 (0.6)	4.6 (0.6)
Phosphate mg/dL	1.3 (0.3)	1.4 (0.3)	1.3 (0.3)	1.3 (0.3)
Urea mmol/L	22.4 (10.3)	19.9 (6.9)	24.2 (13.8)	21.8 (9.4)
Albumin g/L	38 (5.0)	37 (4.8)	38 (5.7)	37 (5.8)
Cholesterol mmol/L	4.6 (1.4)	4.6 (1.2)	4.4 (1.2)	4.6 (1.2)
Comorbidity				
Charlson comorbidity index	6.9 (1.8)	7.2 (2.0)	7.1 (1.9)	7.3 (2.0)
Diabetes mellitus	81 (42)	44 (39)	131 (47)	99 (43)

Cerebrovascular disease	27 (14)	30 (26)	91 (32)	65 (28)
Coronary artery disease	60 (31)	18 (16)	53 (19)	43 (19)
Malignancy	33 (17)	24 (21)	61 (22)	61 (27)
Heart failure	38 (20)	22 (19)	48 (17)	47 (21)
Education				
Low	33 (21)	33 (31)	79 (32)	60 (29)
Intermediate	87 (54)	53 (51)	123 (49)	104 (51)
High	31 (19)	18 (17)	40 (16)	32 (16)
Other	9 (6)	1 (1)	7 (3)	9 (4)
Marital status				
Married/partner	113 (71)	61 (58)	175 (71)	133 (65)
Divorced/widowed/single	47 (29)	44 (42)	72 (29)	71 (35)
Lifestyle				
Smoking				
Never	61 (32)	38 (37)	96 (39)	76 (37)
Current smoker	89 (46)	54 (52)	134 (55)	100 (49)
Former smoker	8 (4)	12 (11)	15 (6)	28 (14)
Alcohol consumption				
No consumption	75 (50)	55 (54)	139 (58)	110 (55)
1-4 standard units/week	46 (30)	23 (23)	64 (27)	55 (27)
>4-7 standard units/week	15 (10)	10 (10)	22 (9)	14 (7)
>7 standard units/week	15 (10)	13 (13)	16 (6)	2 (11)1

*the low-protein diet (LPD) exposure was measured as a time-varying covariate. A patient who is in the LPD group could have been either in any of the LPD groups from baseline, or in any of the standard diet groups at baseline. Thus, the groups above are not mutually exclusive.

Table S3. Adjusted risk of mortality and decline in subjective global assessment associated with a prescribed low-protein diet, according to subgroups.

**MORTALITY**			
	**Adjusted OR (95% CI)**	**Adjusted OR (95% CI)**	**p-value interaction**
**Age**	**Age ≤75 years**	**Age > 75 years**	
Standard diet	Ref.	Ref.	
Low protein diet ≤0.8 g/kg	0.88 (0.55-1.42)	1.37 (0.94-1.98)	<0.001
Low protein diet ≤0.6 g/kg	0.77 (0.43-1.36)	1.18 (0.79-1.77)	0.02
**Sex**	**Women**	**Men**	
Low protein diet ≤0.8 g/kg	n/a	n/a	0.19
Low protein diet ≤0.6 g/kg	n/a	n/a	0.91
**Subjective global assessment**	**SGA score <6**	**SGA score 6-7**	
Standard diet	Ref.	Ref.	
Low protein diet ≤0.8 g/kg	1.01 (0.73-1.41)	1.74 (0.93-3.127)	0.04
Low protein diet ≤0.6 g/kg	n/a	n/a	0.16
**Comorbidity**	**Charlson comorbidity index score ≤6**	**Charlson comorbidity index score >6**	
Standard diet	Ref.	Ref.	
Low protein diet ≤0.8 g/kg	0.76 (0.45-1.30)	1.34 (0.94-1.90)	<0.001
Low protein diet ≤0.6 g/kg	0.64 (0.33-1.23)	1.15 (0.79-1.68)	0.003
**Diabetes mellitus**	**Diabetes**	**No diabetes**	
Standard diet	Ref.	Ref.	
Low protein diet ≤0.8 g/kg	n/a	n/a	0.09
Low protein diet ≤0.6 g/kg	n/a	n/a	0.27
**DECLINE IN NUTRITIONAL STATUS**			
	**Adjusted OR (95% CI)**	**Adjusted OR (95% CI)**	**p-value interaction**
**Age**	**Age ≤75 years**	**Age > 75 years**	
Standard diet	Ref.	Ref.	
Low protein diet ≤0.8 g/kg	0.93 (0.48-1.83)	1.16 (0.69-1.93)	0.03
Low protein diet ≤0.6 g/kg	1.07 (0.54-2.13)	1.54 (0.89-2.66)	0.004

Sex	Women	Men	
Low protein diet ≤0.8 g/kg	n/a	n/a	0.25
Low protein diet ≤0.6 g/kg	1.47 (0.66-3.32)	1.38 (0.84-2.27)	0.01
**Diabetes**	**Diabetes**	**No diabetes**	
Low protein diet ≤0.8 g/kg	n/a	n/a	0.21
Low protein diet ≤0.6 g/kg	1.40 (0.80-2.45)	1.43 (0.75-2.75)	0.04
**Subjective global assessment**	**SGA score <6**	**SGA score 6-7**	
Low protein diet ≤0.8 g/kg	n/a	n/a	0.93
Low protein diet ≤0.6 g/kg	n/a	n/a	0.14
**Comorbidity**	**Charlson comorbidity index score ≤6**	**Charlson comorbidity index score >6**	
Standard diet	Ref.	Ref.	
Low protein diet ≤0.8 g/kg	1.00 (0.47-2.12)	1.05 (0.63-1.75)	0.01
Low protein diet ≤0.6 g/kg	0.96 (0.45-2.03)	1.49 (0.87-2.54)	<0.001

Table S4. Sensitivity analyses for the risk of mortality associated with a low protein diet

**Prescribed diet**	**Unadjusted OR**	**Adjusted OR***
**Imputed missing laboratory values**		
Standard diet	ref	ref
Low protein diet ≤0.8 g/kg	1.11 (0.93-1.35)	1.00 (0.78-1.27)
**Imputed missing laboratory values**		
**Measured diet (ideal BW)**	Unadjusted OR	Adjusted OR*
Standard diet, adherent	Ref.	Ref.
Low protein diet ≤0.8 g/kg, adherent	1.49 (1.03-2.16)	0.94 (0.57-1.53)¤
Low protein diet ≤0.8 g/kg, non-adherent	1.59 (1.07-2.35)	1.10 (0.73-1.64)
Standard diet, spontaneously low protein intake	Ref.	Ref.
Low protein diet ≤0.8 g/kg, adherent	1.04 (0.71-1.53)	0.98 (0.58-1.68)
**According to actual bodyweight**		
Standard diet, adherent	Ref.	Ref.
Low protein diet ≤0.8 g/kg, adherent	1.33 (0.91-1.94)	1.01 (0.58-1.77)
Low protein diet ≤0.8 g/kg, non-adherent	1.23 (0.80-1.87)	0.81 (0.40-1.60)
Standard diet, spontaneously low protein intake	Ref.	Ref.
Low protein diet ≤0.8 g/kg, adherent	1.16 (0.83-1.61)	1.24 (0.75-2.04)

¤dropped spline and country in outcome model to make the model converge
